# Text-mining-based feature selection for anticancer drug response prediction

**DOI:** 10.1093/bioadv/vbae047

**Published:** 2024-03-26

**Authors:** Grace Wu, Arvin Zaker, Amirhosein Ebrahimi, Shivanshi Tripathi, Arvind Singh Mer

**Affiliations:** Division of Engineering Science, University of Toronto, Toronto, M5S2E4, Canada; Department of Biochemistry, Microbiology & Immunology, University of Ottawa, Ottawa, K1H8M5, Canada; Ottawa Institute of Systems Biology, University of Ottawa, Ottawa, K1H8M5, Canada; Department of Biochemistry, Microbiology & Immunology, University of Ottawa, Ottawa, K1H8M5, Canada; Department of Biochemistry, Microbiology & Immunology, University of Ottawa, Ottawa, K1H8M5, Canada; Ottawa Institute of Systems Biology, University of Ottawa, Ottawa, K1H8M5, Canada; Department of Biochemistry, Microbiology & Immunology, University of Ottawa, Ottawa, K1H8M5, Canada; Ottawa Institute of Systems Biology, University of Ottawa, Ottawa, K1H8M5, Canada; School of Electrical Engineering & Computer Science, University of Ottawa, Ottawa, K1N6N5, Canada

## Abstract

**Motivation:**

Predicting anticancer treatment response from baseline genomic data is a critical obstacle in personalized medicine. Machine learning methods are commonly used for predicting drug response from gene expression data. In the process of constructing these machine learning models, one of the most significant challenges is identifying appropriate features among a massive number of genes.

**Results:**

In this study, we utilize features (genes) extracted using the text-mining of scientific literatures. Using two independent cancer pharmacogenomic datasets, we demonstrate that text-mining-based features outperform traditional feature selection techniques in machine learning tasks. In addition, our analysis reveals that text-mining feature-based machine learning models trained on *in vitro* data also perform well when predicting the response of *in vivo* cancer models. Our results demonstrate that text-mining-based feature selection is an easy to implement approach that is suitable for building machine learning models for anticancer drug response prediction.

**Availability and implementation:**

https://github.com/merlab/text_features.

## 1. Introduction

High-throughput genomic technologies have enhanced our knowledge of the molecular origins of cancer and have contributed toward the personalized cancer treatment (Lightbody *et al.* 2019, Gambardella *et al.* 2020). Given the complexity and heterogeneity of cancer, comprehensive studies across many tumor types and drugs are required for the effective translation of personalized medicine to clinics. To fulfill the exceeding demand of cancer pharmacogenetic data, several large-scale projects have been conceived. Projects such as the Cancer Cell Line Encyclopedia (CCLE) (Barretina *et al.* 2012) and Genomics of Drug Sensitivity in Cancer (GDSC) (Garnett *et al.* 2012) provide genomic data from diverse cancer cell lines along with drug sensitivity data of anticancer drugs tested on these cell lines. These large-scale datasets have enabled the use of machine learning methods to build computational models that can predict drug response from genomic data (Geeleher *et al.* 2014, Ammad-ud-din *et al.* 2016, Suphavilai *et al.* 2018, Ali and Aittokallio 2019). However, gene expression profiles contain a large number of features (approximately 22 000 protein coding genes). Training on such a huge number of features is time-consuming, computationally intensive and prone to producing noisy estimates (Liu and Motoda 1998). Selection of important variables and elimination of unessential ones is of tremendous importance for the machine learning model building process as it reduces the complexity of the problem at hand, aids in debugging, enhances model interpretation, and speeds up the training process. Machine learning models trained on selected features are less resource intensive and can lead to higher accuracy (Nilsson *et al.* 2007, He and Yu, 2010, Kirpich *et al.* 2018, Degenhardt *et al.* 2019).

Feature selection methods fall into three main categories: wrappers, embedded methods, and filtering techniques (Chandrashekar and Sahin 2014, Pudjihartono *et al.* 2022). Wrappers treat feature selection as a search problem, constructing and evaluating machine learning models with varying feature subsets. They engage in an exhaustive “wrap-around” process to identify the most suitable features, utilizing methods like forward selection and backward elimination. Despite their effectiveness, wrappers can be computationally demanding due to their exhaustive search across feature combinations. Embedded methods integrate feature selection within model training, considering feature importance as part of the algorithm. Examples include techniques such as LASSO regression penalizes complex models, favoring simpler ones with fewer but more informative features. These methods are efficient and less computationally intensive compared to wrappers. Filtering methods rely on statistical measures to rank and select features based on their individual relevance and redundancy. They use techniques like correlation or mutual information-based selection. Additionally, methods like MRMR (Minimum Redundancy Maximum Relevance) aim to select highly relevant features while minimizing redundancy (Radovic *et al.* 2017). The efficiency of these methods can range from highly efficient, like correlation, to more resource-intensive, like MRMR. Recent advancements in deep learning have revolutionized feature selection by employing internal mechanisms for automatic feature extraction and selection. Models use techniques such as L1 regularization to implicitly identify important features. However, the “black box” nature of these models can pose challenges in understanding feature contributions. To address this, hybrid approaches are emerging, combining traditional methods with deep learning models. For instance, filters pre-select a smaller feature set, which is then refined using deep learning models. Genetic algorithm (Wang *et al.* 2023) (GA) and embedded-wrapper hybrids are some of the examples of the hybrid feature selection approaches.

Due to the significance of feature selection in drug response prediction, several methods have been proposed (Dong *et al.* 2015, Ali and Aittokallio 2019, Xu *et al.* 2019, Ahmed *et al.* 2020, Emdadi and Eslahchi, 2021, Ba-Alawi *et al.* 2022). Recursive feature selection (Dong *et al.* 2015), network-based feature selection (Ahmed *et al.* 2020), autoencoder with Random Forest (Xu *et al.* 2019), or Hidden Markov models (Emdadi and Eslahchi 2021) are example of some of the proposed feature selection methods. However, such data driven methods are time consuming, complex to implement and computationally resource intensive.

**Figure 1.**
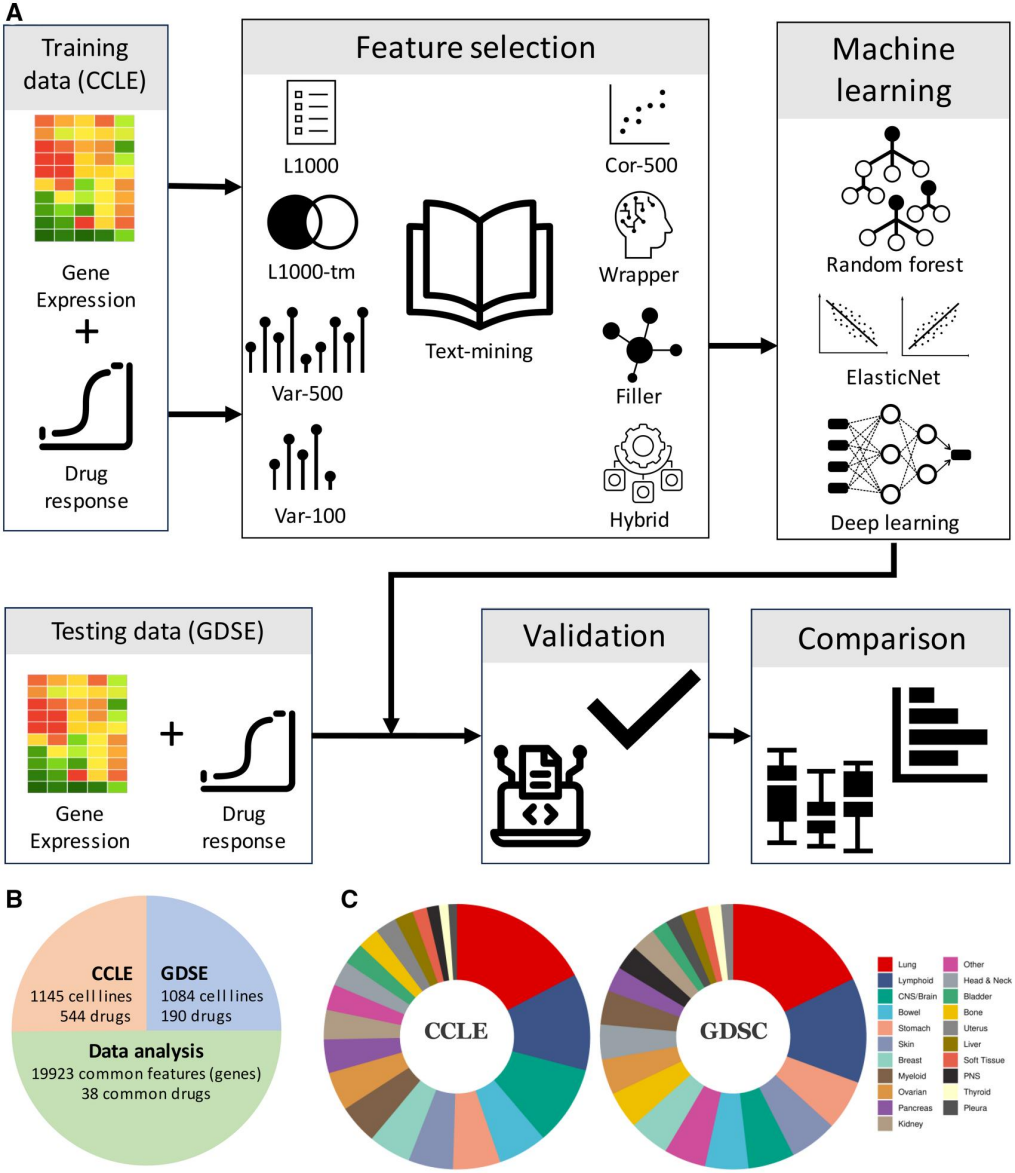
Methodology of the article and description of datasets. (A) CCLE dataset was used for feature selection and model training. Nine different feature selection methods were applied. For each feature selection method, we trained three machine learning models to predict drug response from gene expression data. Validation was performed using the GDSE dataset and results were used to compare various feature selection methods. (B) Summary of sources of the datasets, number of features and drugs analyzed in this study. (C) Distribution of the tissue of origin types in the CCLE and GDSE datasets.

In this article, we propose a text-mining-based feature selection approach that takes advantage of the large amount of peer-reviewed scientific literature related to the drugs. Academic literature is abundantly available online and can be used as a resource to identify gene-drug connection. This allowed us to prioritize genes (features) for pharmacogenomic machine learning analysis. Using the univariate analysis, we showed that the text-mining-based features have higher association with drug response. Next, we evaluated the text-mining-based features selection approach using multiple machine learning methods including Elastic Net, Random Forest, and deep-learning approach for drug response prediction (Fig. 1A). Results show that the text-mining-based feature selection strategy provides superior features and significantly improves the performance of machine learning models for drug response prediction.

**Figure 2.**
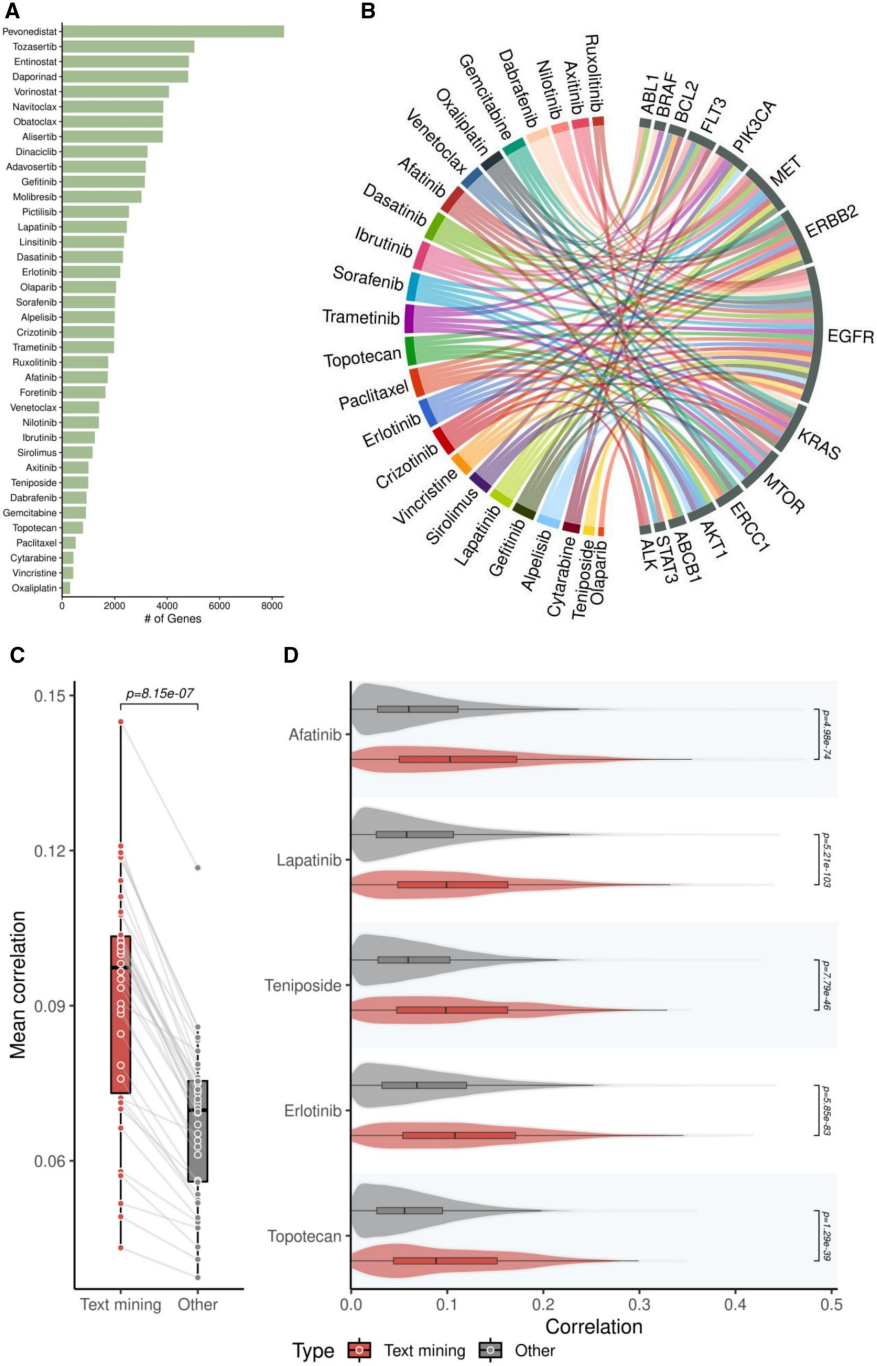
Text-mining-based genes have higher correlation with drug response. (A) Distribution of genes associated with anticancer drugs in the scientific literature. The x-axis represents the number of genes associated with a drug (y-axis) in literature. (B) Genes that frequently associated with drugs are shown in circular visualization. (C) Correlation between text-mining based and drug response in cancer pharmacogenomic datasets. Each point in the boxplot represents a drug. Red boxplot represents average correlation value between drug response and expression of text-mining-based features for a given drug. Text-mining-based features show a higher correlation with drug response in comparison with other features (paired *t*-test *P*-value= 8.1e-07). (D) Correlation be-tween gene expression features (text-mining in red and others in gray) and drug response (AAC) for drugs. Student’s *t*-test-based *P*-values are shown for each drug.

## 2 Methods

### 2.1 Data

In our investigation, we used the Cancer Cell Line Encyclopedia (CCLE) (Barretina *et al.* 2012) dataset for training, and the Genomics of Drug Sensitivity in Cancer (GDSCv2) (Garnett *et al.* 2012, Iorio *et al.* 2016) dataset for testing. The CCLE dataset has 1145 cell lines and the GDSC dataset has 1084 cell lines. These datasets were obtained from the Orcestra platform (Mammoliti *et al.* 2021). We focused our analysis on 38 FDA approved common drugs between the two datasets (Fig. 1B and C and Supplementary Fig. S1). Following the guidelines proposed by Sharifi-Noghabi *et al.* (2021), we defined the drug response as the area above the drug-dose response curve (AAC). R tools PharmacoGx (Smirnov *et al.* 2016) and Xeva (Mer *et al.* 2019) were used for *in vitro* and *in vivo* pharmacogenomics data analysis.

### 2.2 Feature selection

We assessed the performance of text-mining-based feature selection procedure against eight other feature selection approaches (Table 1). The text-mining-based features for each drug were obtained from the Génie web application (Fontaine *et al.* 2011). A comprehensive description of the Génie algorithm for selecting drug-associated genes can be found in Supplementary Methods section and illustrated in Supplementary Fig. S2. In brief, given a specific topic, Génie algorithm searches the MEDLINE database for related abstracts and trains a naive Bayes linear classifier on 1000 abstracts (Fontaine *et al.* 2011). Using this model, the algorithm classifies articles associated with each gene and assigns a *P*-value using Fisher’s exact test. Based on a *P*-value cutoff (default *P* < .01), the algorithm outputs a list of relevant genes sorted by increasing false discovery rate. Complete lists of text-mining-based genes (features) are available in Supplementary Data File S1. Selecting features based on their variance is a commonly used unsupervised feature selection method (Guyon and Elisseeff 2003). We used the top 100 and 500 features by variance, annotated as var-100 and var-500 respectively in the analysis. We also tested correlation-based supervised feature selection. In the training data, Pearson correlation coefficient was computed between each feature and drug response. The top 500 genes show the highest correlation with drug response were selected as features for training the machine learning model. Landmark-1000 (L1000) gene set is known to be reproducible and capable of inferring expression levels of the majority of other genes (Subramanian *et al.* 2017, Jeon *et al.* 2022). This gene set is frequently used for characterizing biological samples (Malta *et al.* 2018, Wan *et al.* 2020) and machine learning-based drug response prediction (Gardiner *et al.* 2020, Lu *et al.* 2021, Uner *et al.* 2023). We included the L1000 gene-set as features in our analysis. For drugs, the L1000 gene-set may show overlap with text-mining based genes, therefore, for each drug, we created a separate feature set, by removing text-mining genes from the L1000 gene-set called the L1000-tm feature set. To ensure a comprehensive coverage of feature selection approaches, we incorporated three additional techniques. The recursive feature elimination (RFE) method was chosen as a representative wrapper method of feature selection. Additionally, our study incorporated the MRMR (Minimum Redundancy Maximum Relevance) technique, a widely utilized filter-based feature selection approach (Radovic *et al.* 2017). Finally, a GA-based feature selection method was employed to represent hybrid feature selection approaches.

**Table 1.** Description of candidate feature selection methods.

Feature selection method	Description
text-mining	Feature (genes) set obtained using text-mining of scientific literature
var_100	Feature set containing top 100 genes by variance in the training data
var_500	Feature set containing top 500 genes by variance in the training data
cor_500	Set of 500 genes with the highest Pearson correlation with the output variable
L1000	Feature set containing all genes from L1000 gene list
L1000-tm	Feature set containing genes from L1000 gene list, except those that appear in the text-mining feature list are removed.
MRMR	Features selected using the MRMR (minimum redundancy maximum relevance) algorithm on the training set.
GA	Features selected by applying the genetic algorithm on the training dataset
RFE	Features selected through recursive feature elimination algorithm on the training set

### 2.3 Univariate analysis

We conducted univariate analysis by calculating the absolute value of the Pearson correlation between the level of expression for each gene, and AAC. This analysis was performed for each drug, using the corresponding text-mining genes and the other remaining genes. The significance of the difference between text-mining and non-text mining genes were assessed using Student’s *t*-test.

### 2.4 Training and testing machine learning models

The goal of multivariate analysis in this problem is to predict drug sensitivity using the expression of selected genes. Drug sensitivity was defined using the area above the drug-dose response curve (AAC) and can have a continuous value between 0 and 1. Therefore, regression machine learning analysis was performed to train the models. We applied Elastic Net (Friedman *et al.* 2010, Tay *et al.* 2023) and Random Forest (Breiman 2001, Touw *et al.* 2013) and deep learning (Sakellaropoulos *et al.* 2019) for the multivariate analysis. The elastic net model is a regularized regression, combining penalties of ridge and lasso regression approaches (Tay *et al.* 2023). The Random Forest model is an ensemble of decision trees, each trained on a subset of training data (Touw *et al.* 2013). During testing, the final output is determined by averaging predictions from all the trees. We employed Elastic Net and Random Forest models to compare nine distinct feature selection algorithms, as detailed in Table 1.

In recent years, deep neural network-based approaches have gained popularity due to their enhanced capability to model biological complexity (Sakellaropoulos *et al.* 2019, Kuenzi *et al.* 2020, Baptista *et al.* 2021, Chen and Zhang 2022). We employed the TensorFlow library, a well-known deep-learning framework, to construct and train our network. Our network architecture, regardless of the specific feature set, employs stacked fully connected layers. For the hidden layers, Rectified Linear Unit (ReLU) activation functions were utilized, while the output layers employed sigmoid activation. The use of sigmoid activation for the output layer ensures that the predicted drug response values are confined within the range of 0–1, consistent with expected values. To optimize our models, we employed stochastic gradient descent with a learning rate of 0.01 and a momentum of 0.9. The mean-squared error (MSE) chosen as a loss function. Additionally, we implemented dropout regularization after each hidden layer with a dropout rate of 20% to mitigate overfitting. Similar to other machine learning tasks, we used cross-validation for parameter optimization. Each model underwent 15 epochs, iterated six times in the process. During training, the scikit-learn library facilitated cross-validation, enabling the selection of the best models for validation purposes. Employing deep learning method, we evaluated six unique feature selection algorithms: var-100, var-500, L1000, L1000-tm, cor-500, and text-mining (Table 1).

### 2.5 Training and evaluation

The data were processed by removing samples with missing values and low variance between genes. We removed samples that contain zero variance for more than 85% genes. For each model type, we performed 4-fold cross-validation that was repeated 20 times, each time with different resample indices. The train-test split was 80/20 and hyperparameter tuning was performed using grid-search. The relevant feature selection method was applied on the dataset, after which the input features were scaled and centered by dividing by the standard deviation and subtracting the mean of the data, respectively. In the multivariate analysis, we measured the performance using the Pearson correlation, Spearman's rank correlation coefficient, Kendall's Tau, root-mean-squared error (RMSE), MSE, and mean absolute error (MAE) that are reported in Supplementary Data File S2. Both within-domain and cross-domain analysis were performed with the CCLE training dataset and the GDSCv2 testing dataset. For cross-domain validation, the model with the highest performance was chosen and the validation correlation was reported.

### 2.6 Validation on patient-derived xenograft data

We conducted validation based on patient-derived xenografts (PDX) using the Novartis PDX Encyclopedia (PDXE) dataset (Gao *et al.* 2015). Drug erlotinib was selected due to its presenccross three datasets: CCLE-CTRPv2, GDSC, and PDXE. Leveraging the R package Xeva (Mer *et al.* 2019) (version 1.18.0), we extracted gene expression and PDX-related drug response data for erlotinib. This cohort contains gene expression and erlotinib response on PDXs from 23 non-small cell lung cancer patients. To ensure coherence between datasets, we curated a subset of gene expression features present in both CCLE-CTRPv2 and PDXE datasets. With these shared text-mining features, we trained a Random Forest model on the CCLE-CTRPv2 dataset for the drug erlotinib. Upon optimization, this model was deployed to predict drug response using the PDXE gene expression data. The predicted drug responses were dichotomized into high and low groups based on the median values. We used Kaplan–Meier (KM) plots and conducted log-rank tests for statistical analysis to discern the difference between high and low groups.

## 3 Results

### 3.1 Text-mining-based genes are highly correlated with drug response

To assess the usefulness of text-mining-based features for drug response prediction, we first mined the genes associated with drugs in scientific literature. Analysis was done for 38 different drugs that were present in both CCLE-CTRPv2 and GDSC datasets (Fig. 2A and Supplementary File S1). We observed a large variation in the number of text-mining-based genes associated with different drugs (Fig. 2A and B). Drugs such as oxaliplatin and paclitaxel had low number of text-mining-based genes (307 and 518, respectively, for oxaliplatin and paclitaxel), while drug such as tozasertib and pevonedistat had very high number of genes associated (5043 and 8456, respectively, tozasertib and pevonedistat). Genes such as EGFR, ERBB2, TP53 and MAPK family genes were associated with high number of drugs (Fig. 2B and Supplementary Fig. S3). Furthermore, EGFR gene was among the top 10 genes for 32 different drugs. Next, we explored the potential of text-mining-based genes as drug biomarkers. For this we computed the association between the response of a drug on cell-lines and gene expression values using correlation. Our results show that the text-mining features (genes) have significantly higher correlation with drug response when compared against other features (Fig. 2C, pairwise *t*-test *P *=* *8.18 × 10^−7^). Multiple tyrosine kinase inhibitors such as erlotinib, lapatinib, and afatinib were among the top drugs for which text mining features show high correlation to the drug response (Fig. 2D, *t*-test *P *=* *5.8 × 10^−83^, 5.5 × 10^−103^, and 4.9 × 10^−74^ for erlotinib, lapatinib, and afatinib, respectively). Gene overrepresentation analysis revealed the enrichment of drug-relevant pathways within the text-mining-based features (see Supplementary results section and Supplementary Figs S4–S7). Collectively, these results indicate that the text-mining-based genes have higher correlation with drug activity.

### 3.2 Text-mining-based genes perform better in machine learning tasks

**Figure 3.**
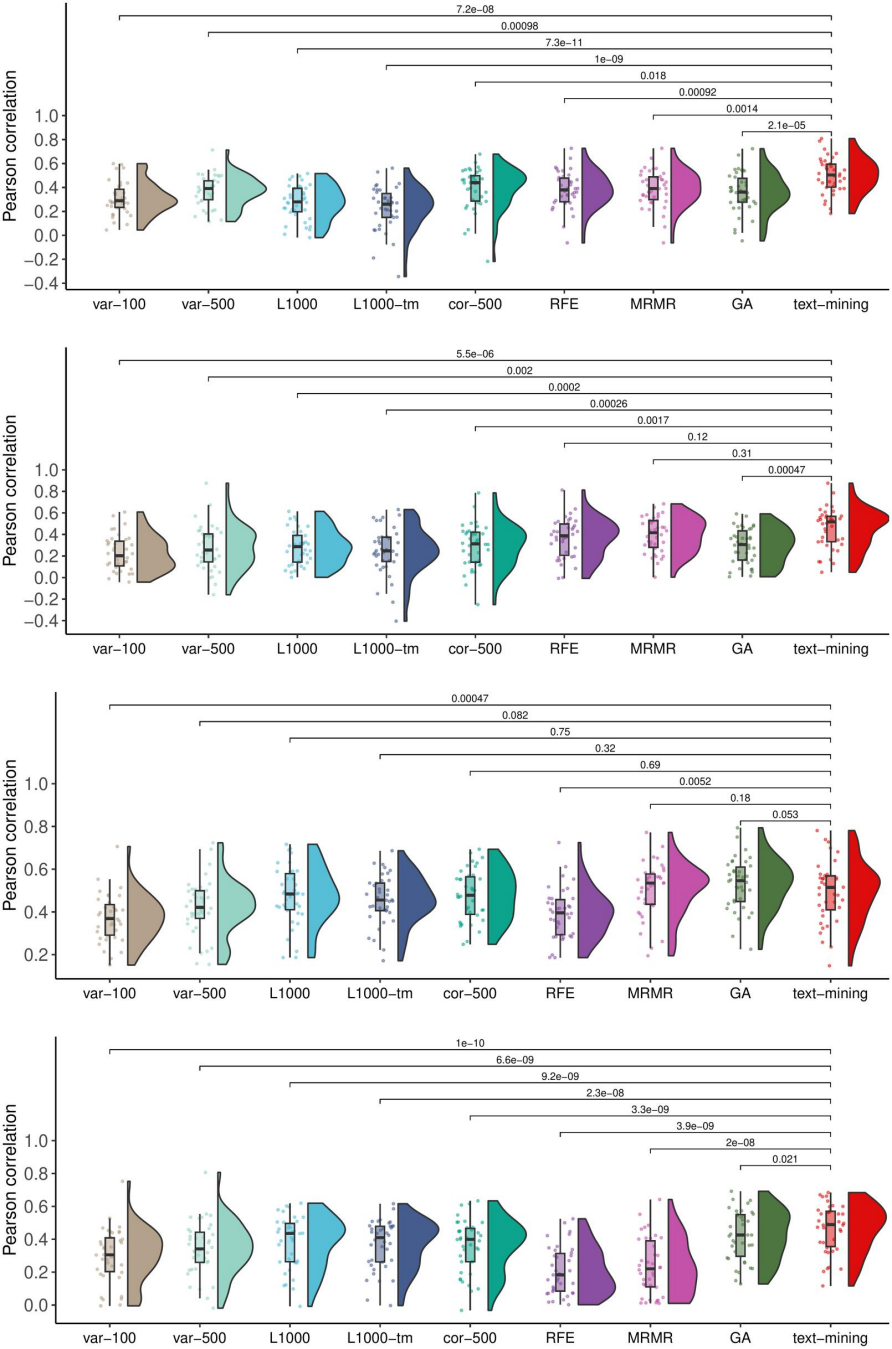
Text-mining-based features perform better in machine learning task of drug response prediction. (A) Training and (B) validation of drug response prediction models using Random Forest algorithm. (C) and (D) represent training and validation of drug response prediction models using Elastic Net algorithm. In the rain-cloud plots, each dot represents a drug, x-axis represents different feature selection approaches and y-axis represents correlation between response of a drug and predicted response value. Student’s *t*-test used for *P*-values computation.

**Figure 4:**
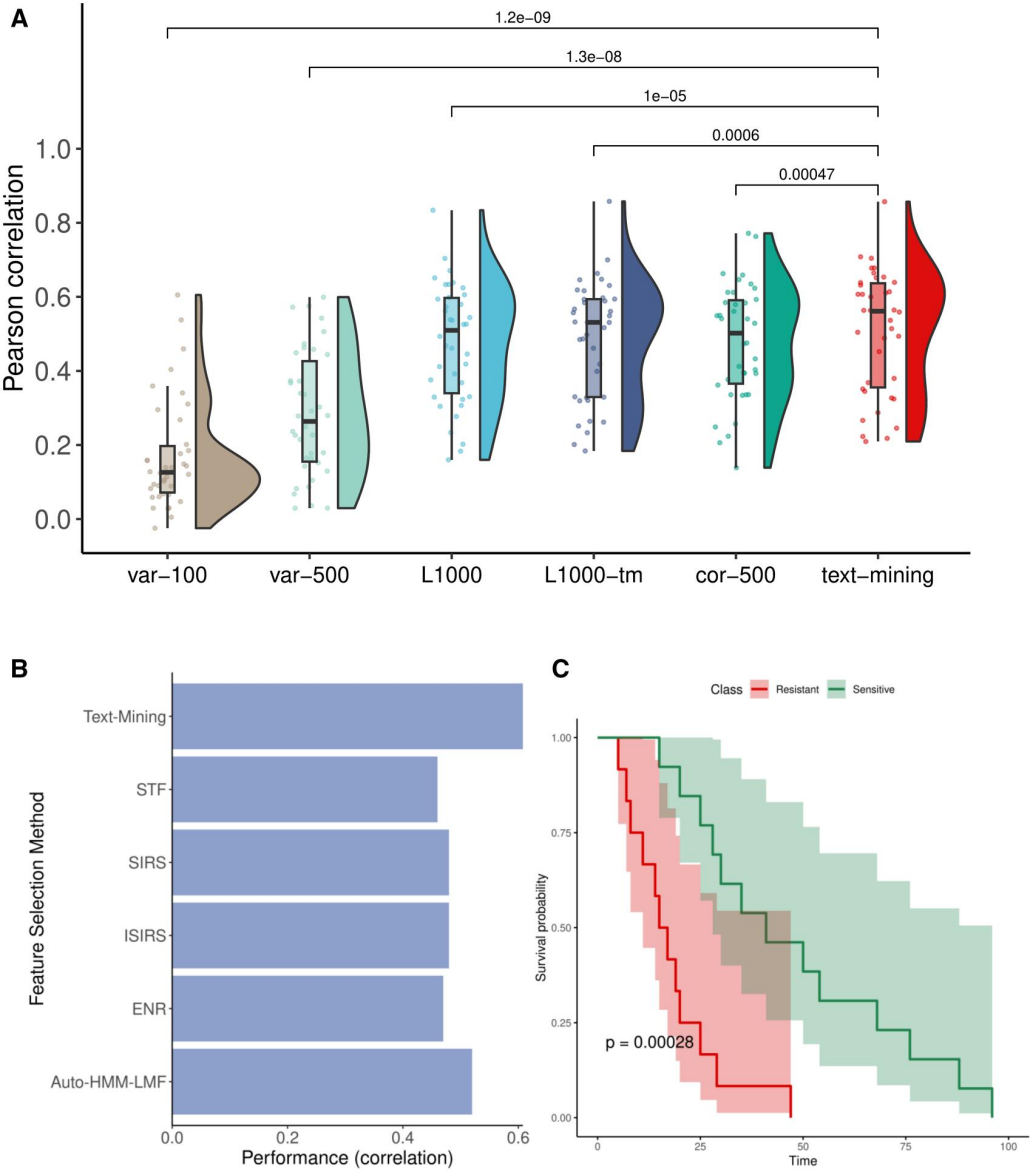
Validation of text-mining-based features selection approach using deep-learning and external dataset. (A) Performance of deep learning models of different feature selection approach on validation dataset. (B) Comparison of the performance of text-mining-based features to other feature selection approaches. (C) Kaplan–Meier (KM) plot of predicted drug response in (PDXs) using text-mining features. For erlotinib, a random forest model was trained using text-mining genes on the in vitro data. This model was used for predicting sensitivity of erlotinib in PDXs. Statistically significant difference was observed between labels predicted using text-mining features (log-rank test *P *=* *.0002).

Feature selection is a key step in training multivariate machine learning models. To assess if genes selected using text-mining can be utilized as features for machine learning model training, we performed the within-domain and cross-domain testing (Fig. 3). Machine learning models were trained and cross-validated on CCLE dataset (within-domain). We utilized the GDSC dataset for external validated (cross-domain). It is important to highlight that we did not use the GDSC dataset during the process of training the machine leaning models. Therefore, GDSC served as an independent validation dataset for the performance evaluation of the machine learning models. For each drug, multivariate machine learning models were trained using Random Forest, Elastic Net and deep learning algorithm. Performance of text-mining feature-based machine learning models were compared against eight other features selection approaches (Table 1). To assess the performance of our machine learning models, we employed a comprehensive set of metrics, including Pearson correlation, Spearman's rank correlation coefficient, Kendall's Tau, RMSE, MSE, and MAE. These values are reported in Supplementary Data File S2. For Random Forest-based within-domain cross-validation, we found that the text-mining genes performed better than other feature selection approaches (Fig. 3A, paired *t*-test *P *=* *7.2 × 10^−8^, 0.0009, 7.3 × 10^−11^, 1 × 10^−9^, 0.018, 0.0009 0.001, and 2.1 × 10^−5^ respectively for var-100, var-500, L1000, L1000-tm, cor-500, RFE, MRMR, and GA). Validation using training independent data (GDSC) shows that text-mining based feature selection approach outperformed var-100, var-500, L1000, L1000-tm, cor-500, and GA (paired *t*-test *P *=* *5.5 × 10^−6^, 0.002, 0.0002, 0.0002, 0.0017, and 0.0004, respectively; Fig. 3B). For Elastic Net based models, we observed that the L1000, cor-500, RFE, and MRMR-based feature sets have equivalent performance to text-mining-based features within-domain analysis (Fig. 3C). However, alidation on independent data shows that the text-mining-based features perform best (Fig. 3D, paired *t*-test *P *=* *1 × 10^−10^, 6.6 × 10^−9^, 9.2 × 10^−9^, 2.3 × 10^−8^, 3.3 × 10^−9^, 3.9 × 10^−9^, 2 × 10^−8^, and 0.021 respectively for var-100, var-500, L1000, L1000-tm, cor-500, RFE, MRMR and GA). Our deep-learning results demonstrate that gene features derived from text mining outperform other feature selection methods (Fig. 4A, paired *t*-test *P *=* *1.2 × 10^−9^, 1.3 × 10^−8^, 1 × 10^−5^, 0.0006, and 0.0004 for var-100, var-500, L1000, L1000-tm, and cor-500, respectively). We also observed that the text-mining feature-based deep-learning models have higher performance compared to Random Forest- or Elastic Net-based models. These observations demonstrated that the features selected using a text-mining approach offers a rapid and efficient approach to achieve high performance in machine learning tasks.

### 3.3 Validation of text-mining feature-based model using *in viv*o system

To further assess the performance of our text-mining-based feature selection approach, we first compared it to other published machine learning models (Fig. 4B). Our results show that the machine learning models train on text-mining-based features outperforms complex feature selection approaches such as Auto-HMM-LMF (Emdadi and Eslahchi 2021), sure independence ranking and screening (SIRS), iterative sure independence ranking and screening (ISIRS)(An *et al.* 2020), simple top features (STF, ranking by marginal Pearson correlation), elastic net regression (ENR) and iterative sure independence screening (ISIS)( Zhu *et al.* 2011, Fang *et al.* 2015). We further validated our text-mining-based feature selection approach using patient derived xenograft (PDXs)-based drug screening dataset. For this purpose, we selected the drug Erlotinib for which sensitivity values are present in both *in vitro* (CCLE) and *in vivo* (PDXE) datasets. Using the text-mining-based features, we trained a Random Forest model using CCLE dataset and predicted erlotinib drug sensitivity in patient-derived xenograft (PDX) system. We found that the machine learning model trained on the text-mining features was able to predict drug response in PDXs (Fig. 4, log-rank test *P*-value=.0002).

## 4 Discussion and conclusions

Predicting the response of anticancer drugs from genomic data is a key problem in multiple fields including preclinical experiments, clinical trial design and personalized cancer therapy. Machine learning methods are capable of predicting drug response from gene expression data (Ammad-ud-din *et al.* 2016, Suphavilai *et al.* 2018, Sharifi-Noghabi *et al.* 2021). However, given the large feature space of high-throughput experiment-based gene expression data, feature selection step is required for efficiently training and improving performance of the machine learning model. Here, we have presented a feature selection approach that exploits drug-gene co-occurrence in the scientific literature.

Our univariate and pathway-level analyses demonstrate the presence of drug biomarkers and pathways within the text-mining-based features (Supplementary Figs S4–S7). Erlotinib, a tyrosine kinase inhibitor primarily used for the treatment of non-small cell lung and pancreatic cancer (Bareschino *et al.* 2007), demonstrated promising results in our analysis. Pathway analysis of its text-mined genes revealed significant enrichment in pathways like EGFR tyrosine kinase Inhibitor resistance, ERBB signaling, and MAPK signaling. Additionally, pathways associated with its target cancers were enriched, underscoring the relevance of our text-mined genes in capturing the drug's action. Lapatinib, targeting EGFR and ERBB2 tyrosine kinase phosphorylation to inhibit cell proliferation (Opdam *et al.* 2012) exhibited significant enrichment in EGFR-related signaling pathways in our pathway analysis results. Paclitaxel, a chemotherapy agent disrupting microtubule growth and inducing apoptosis (Ferlini *et al.* 2009, Kampan *et al.* 2015). Pathway enrichment analysis using paclitaxel-related text-mining genes, display enrichment of pathways related to DNA damage, apoptosis regulation, and microtubule function, echoing its known mechanisms. Tozasertib, an anti-cancer medication influencing mitosis, cytokinesis inhibition via Aurora kinases, and cell death through RIPK-1-dependent necroptosis (Gavriilidis *et al.* 2015, Martens *et al.* 2018), showcased significant enrichment in pathways associated with Aurora and RIPK-1 activity in our analysis of text-mining genes. Overall, the analysis reveals a remarkable alignment between text-mining-based genes and known mechanisms of action for drugs, offering a comprehensive understanding of the pathways underlying their therapeutic effects in cancer treatment.

We have shown that the text-mining genes outperform other approaches of feature selection in machine learning tasks. Text-mining-based feature selection method is simple to implement and not as resource intensive when compared to approaches such as autoencoder, Random Forest, or Hidden Markov model. Furthermore, it provides better accuracy in drug response prediction tasks, along with good accuracy in diverse pharmacogenomic models, making it a suitable feature selection method for anticancer drug response prediction from high-dimensional data. In recent years, deep learning models have been utilized for anticancer drug response prediction (Baptista *et al.* 2021, Sharifi-Noghabi *et al.* 2021, Firoozbakht *et al.* 2022, Partin *et al.* 2023) Our analysis utilizing deep learning approaches demonstrates that text-mining-based features selection can attain high performance within limited computational resources and a short timeframe. We hold the belief that combining the text-mining-based feature selection approach with others, such as MRMR, will enhance accuracy and reduce the training time of deep learning models. We aim to address this in our future work.

A limitation of our methodology is that we did not tune the number of input features in various feature selection algorithms. Future work should consider incorporating a step to optimize the number of features, as this could enhance the performance of the machine learning models. Another limitation of text-mining-based feature selection approach is that the quality of the selected features is heavily dependent upon available scientific literature. As a result, its usefulness is constrained in the setting of novel drug molecules, for which there might be limited scientific literature available. In these situations, one can examine the novel drug's quantitative structure–activity relationship (QSAR) to identify an equivalent existing drug and use its features for model training. Several databases exist that offer information on drug targets, which have been utilized for feature selection (Griffith *et al.* 2017, Wishart *et al.* 2018, Koras *et al.* 2020). However, our analysis revealed that these databases contain only a small number of features (Supplementary Fig. S8). Since these databases depend on manual curation of drug-gene associations, their limited utility in machine learning applications becomes apparent. Text-mining-based feature selection approach reduces reliance on manual feature curation, offering a rapid method for high quality feature selection. Our method involves selecting genes (features) based on their frequency of appearance in published research. Thus, an additional benefit of a text-mining-based feature selection strategy is that it helps open the black box machine learning model by providing biological justification of the selected features (Gardiner *et al.* 2020). From a trained machine learning model, one can determine important features and then consult the associated scientific literature to understand the role of a particular gene in a drug’s mechanism of action. These methods can aid in finding interpretable biomarkers of drug sensitivity and the development of explainable artificial intelligence models. In conclusion, our results show that the text-mining-based feature selection approach can be useful for building machine learning models to predict anticancer drug response.

## Supplementary Material

vbae047_Supplementary_Data

## Data Availability

The data and source code implementing in this article are available at https://github.com/merlab/text_features.
