# Supplementary material for: Text-mining-based feature selection for anticancer drug response prediction
Source: Bioinform Adv. 2024 Mar 26;4(1):vbae047. doi: 10.1093/bioadv/vbae047 (PMC11009020; doi:10.1093/bioadv/vbae047)
Supplement: vbae047_Supplementary_Data [file vbae047_supplementary_data.zip › Supplementary Information.pdf]

Supplementary Information

Text-Mining Based Feature Selection for Anticancer Drug Response Prediction

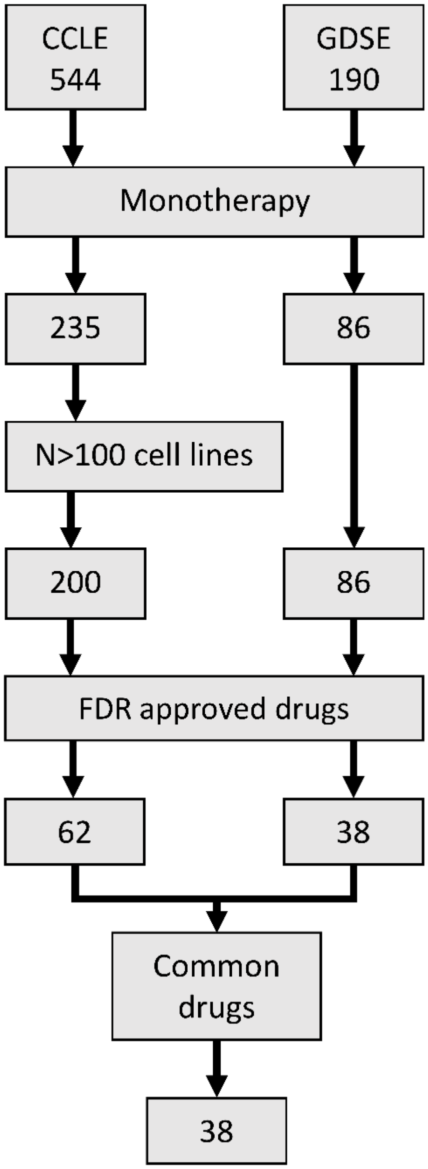

Supplementary figure S1. Filtering steps for the drugs in the study. We filtered for monotherapies from the initial 554 drugs in CCLE and 190 drugs in GDSE datasets. Next, we curated the set of CCLE drugs with at least 100 cell-line information in the dataset. We then identified the drugs that were present in both datasets, yielding 48 common monotherapies. Lastly, we further limited the drug pool to FDA approved drugs, yielding 38 drugs.

Supplementary Method:

The Génie algorithm is a tool developed for gene prioritization across entire genomes in relation to specific topics[1]. In the context of our study, the topic refers to any drug. Here, we describe the working of the Génie algorithm in the context of performing text-mining for drug-related genes (Supplementary Figure S1). The Génie method addresses the challenge of evaluating extensive literature to find genes related to a topic. The Génie algorithm integrates data from several databases, including MEDLINE, NCBI Gene, and HomoloGene. The primary inputs for the algorithm are the name of the species (*Homo sapiens*) and a specific drug (e.g., Paclitaxel). The process begins with retrieving a sample set of MEDLINE abstracts in relation to the drug in question. These abstracts are then used to train a naïve linear Bayesian classifier, which develops a statistical model identifying discriminative words relevant to the drug. Afterward, the algorithm assesses the abstracts linked to the genes of the target species and assigns them a probability score based on the classifier's evaluation. Gene ranking is a crucial component of the Génie algorithm. The algorithm uses a one-sided Fisher's exact test to evaluate whether a gene is significantly associated with a drug. This is done by comparing the number of abstracts that mention each gene with what would be expected in a randomly selected set of abstracts. The algorithm then generates a list of genes in order of their relevance to the specified drug. To summarize, the Génie algorithm uses the literature to identify the connection between genes and drugs. The algorithm employs relevant MEDLINE abstracts to train a naïve linear Bayesian classifier. Using this classifier, it then evaluates all abstracts associated with the genes of the target species and their relevance to the specified drug. We have utilized this methodology for feature selection in our analysis as it provides a list of genes potentially linked to the drug mechanism based on current pharmacology literature.

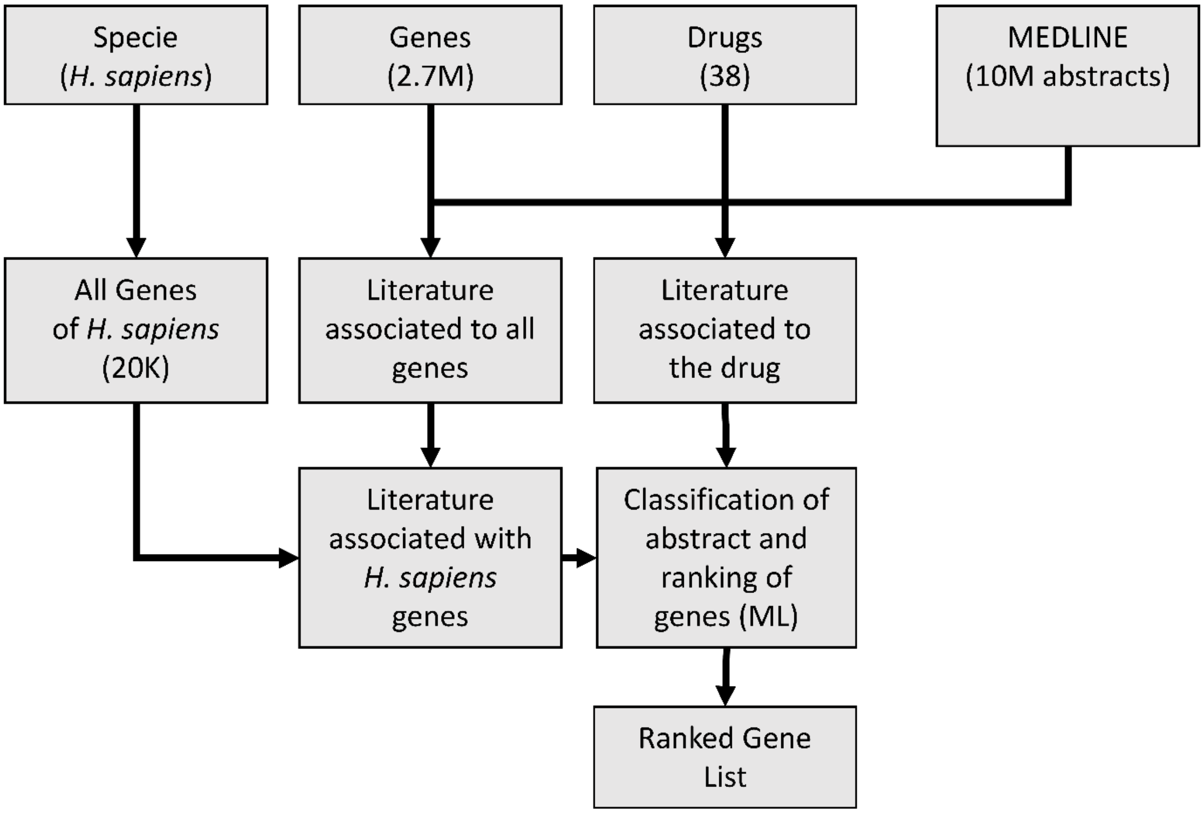

Supplementary figure S2. Summary of the function of Genie for identifying literature-relevant drugs. The genie algorithm uses the species, gene, and drug information, as well as the MEDLINE abstract repository. In the next step, it identifies the literature associated with the drug of interest. Moreover, it identified the literature associated with the genes of *H. sapiens*. In the last step, Génie performs a naïve linear Bayesian classifier followed by a one-tail Fisher Exact test to identify a ranked list of genes associated with the drug of interest.

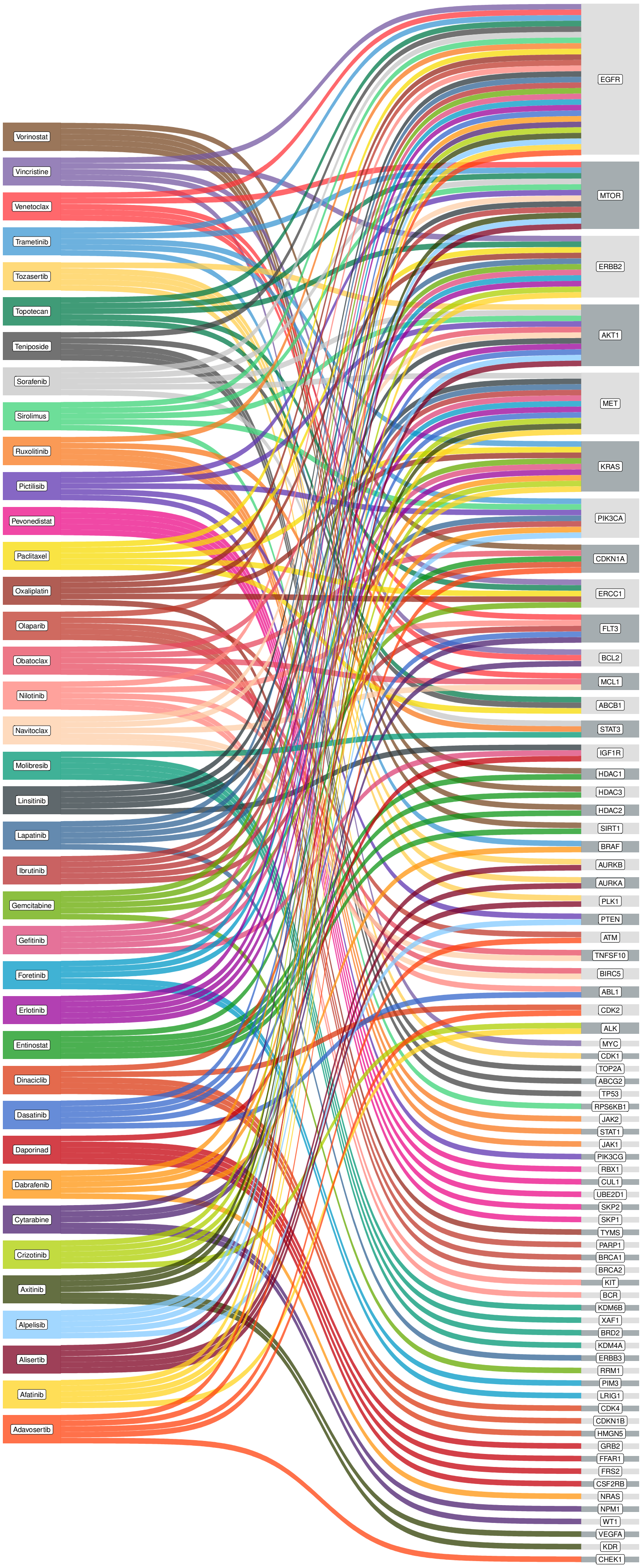

Supplementary figure S3. Sankey plot of the common genes in the text mining datasets. The Genie algorithm was used to identify the genes highly associated with the drug action mechanism based on the literature. Only the top 5 most highly associated genes from each drug are shown.

Supplementary results:

For each of the four drugs below, we used the genes identified through the text-mining algorithm of Genie to perform overrepresentation analysis (ORA). ORA was performed using the “fora” function in the R package `fgsea. We used GO and C2 curated gene sets from MsigDB for the pathway analysis. Overall, Genie algorithm is capable of identifying the genes and pathways associated with the drug action mechanism.

Erlotinib

Erlotinib is a tyrosine kinase inhibitor used primarily in the treatment of non-small cell lung and pancreatic cancer[2,3]. The pathway analysis results of the text mining genes show significant enrichment of pathways like EGFR Tyrosine Kinase Inhibitor Resistance, ERBB Signaling, and MAPK Signaling. Moreover, the pathways associated with non-small cell lung and pancreatic cancers are also significantly enriched. These findings are consistent with Erlotinib's known mechanisms, demonstrating the ability of the Genie algorithm to identify relevant medication in the drug action mechanism (Supplementary Figure-S4).

Lapatinib

Like Erlotinib, Lapatinib targets EGFR and ERBB2 tyrosine kinase phosphorylation, preventing cell proliferation[4,5]. Our pathway analysis results show significant enrichment of EGFR-related signaling pathways in Lapatinib text mining genes, demonstrating the ability of the Genie algorithm to identify relevant medication in the drug action mechanism (Supplementary Figure-S5).

Paclitaxel

Paclitaxel is a chemotherapy drug that interferes with microtubule growth and organization in the cell, hindering cell division and cytokinesis[6–8]. Moreover, the agent binds and deactivates apoptotic inhibitor Bcl-2, thus promoting apoptosis and cell death[9]. The pathway analysis results of the genes from the Genie algorithm show enrichment in DNA damage and apoptotic and microtubule regulation pathways (Supplementary Figure S6).

Tozasertib

Tozasertib is an anti-cancer medication that negatively regulates mitosis and cytokinesis by inhibiting the action of Aurora kinases[10]. Moreover, it promotes cell death through RIPK-1-dependent necroptosis[11]. Our enrichment results of the text mining genes are concordant with the known literature, as we have identified significant enrichment of Aurora and RIPK-1 activity pathways (Supplementary Figure-S7).

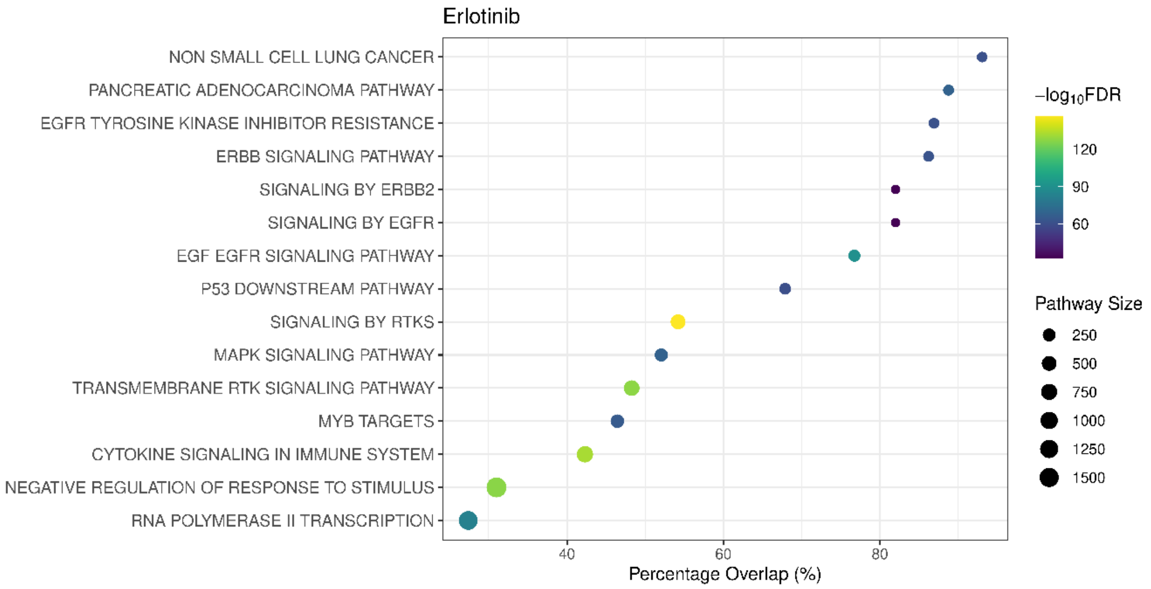

Supplementary figure S4. Over-representation analysis of text-mining genes in pathways databases. Figure shows enrichment of MSigDB pathways in Erlotinib related text-mining genes.

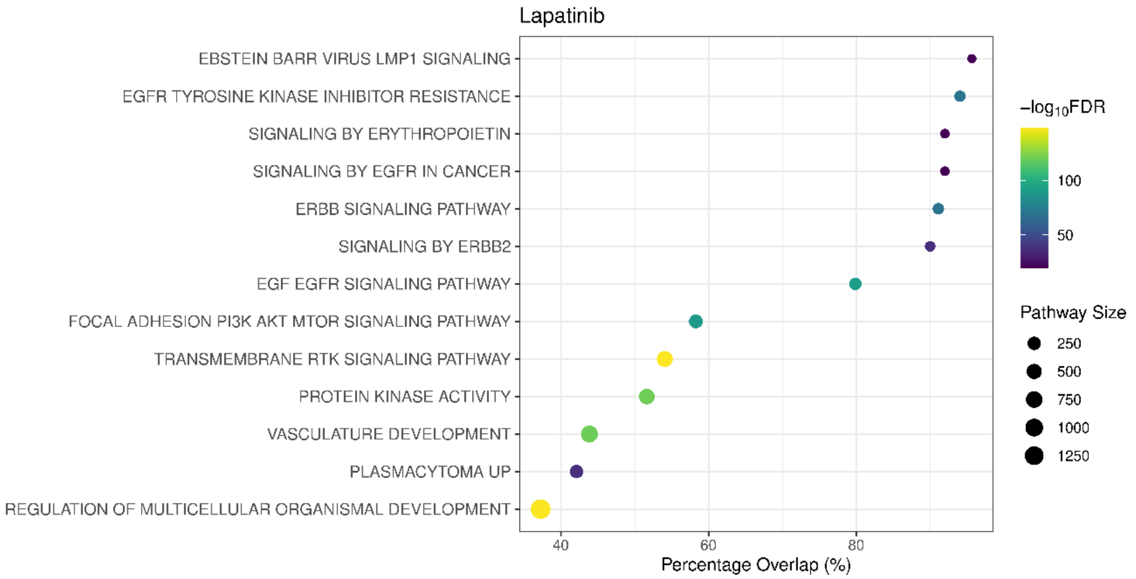

Supplementary figure S5. Over-representation analysis of text-mining genes in pathways databases. Figure shows enrichment of MSigDB pathways in Lapatinib related text-mining genes.

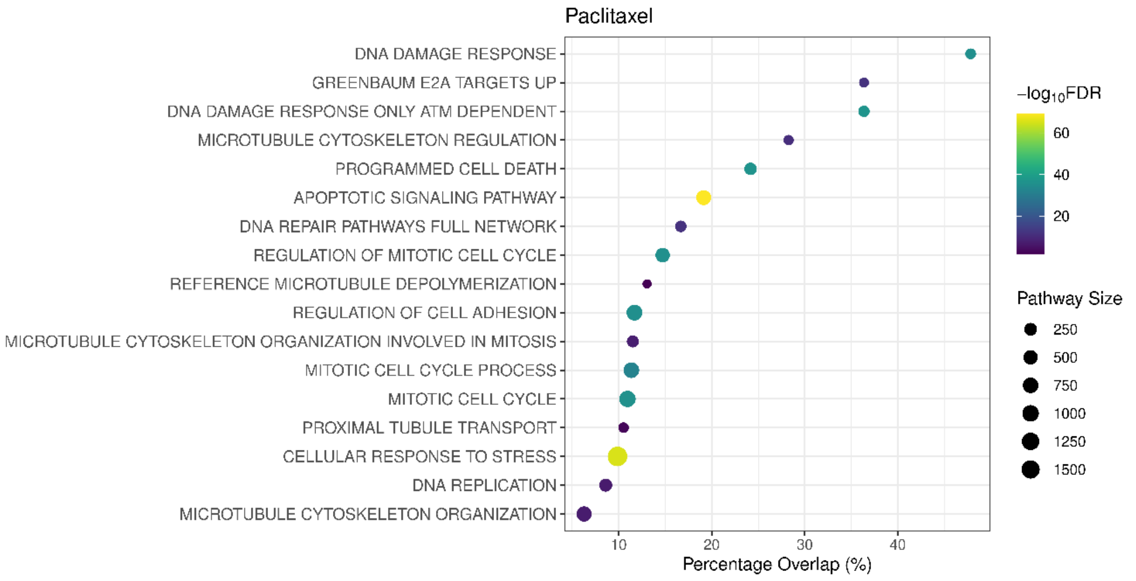

Supplementary figure S6. Over-representation analysis of text-mining genes in pathways databases. Figure shows enrichment of MSigDB pathways in Paclitaxel related text-mining genes.

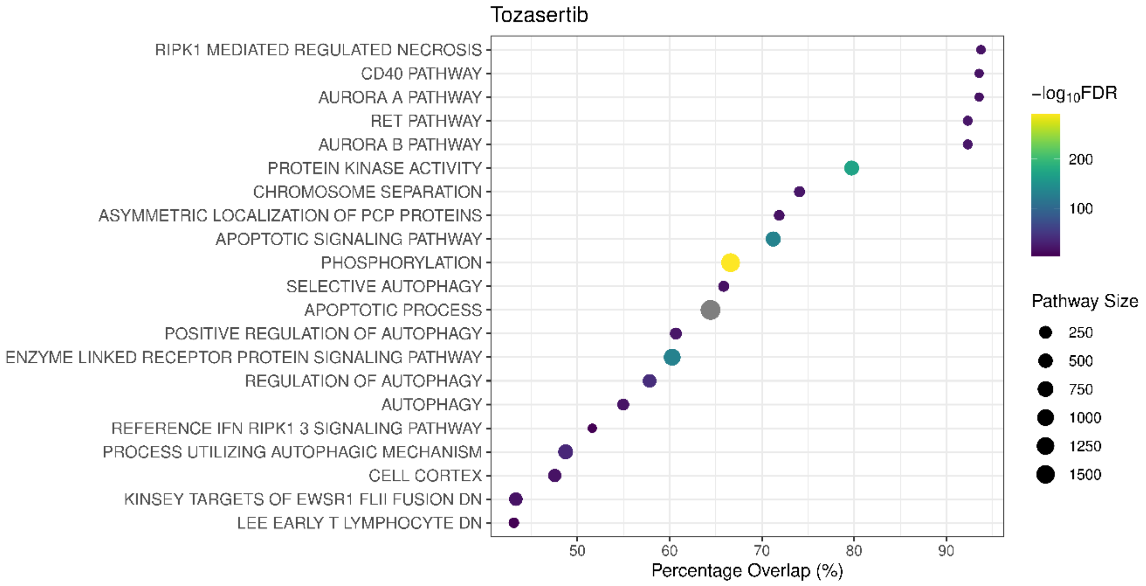

Supplementary figure S7. Over-representation analysis of text-mining genes in pathways databases. Figure shows enrichment of MSigDB pathways in Tozasertib related text-mining genes.

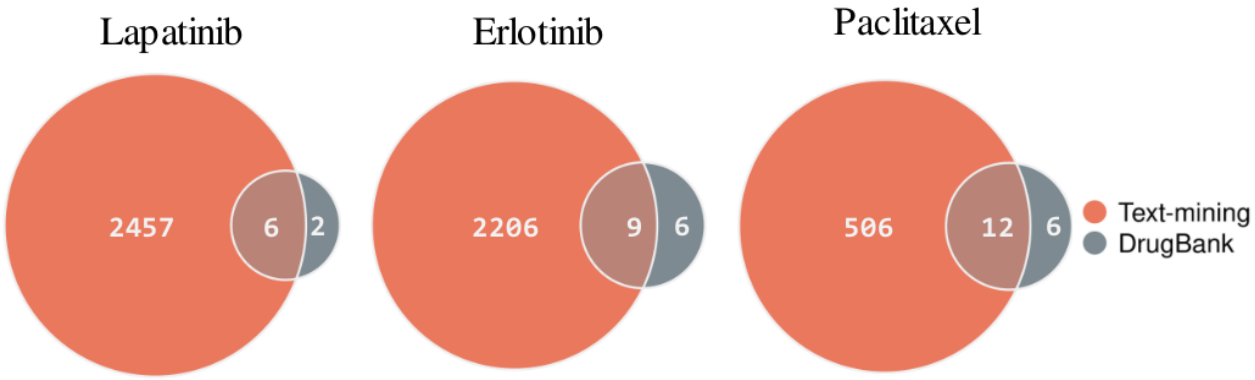

Supplementary figure S8. Venn diagram representing the number of features obtained using text-mining approach and DrugBank database for three different drugs.

Supplementary Table S1. Mean and standard mean errors (SME) of the performance metrics for random forest models based on various feature selection methods. Models with significantly different performance compared to the text-mining method are indicated with * (Student's T-test, $p < 0.05$)

Feature selection method	Pearson	Spearman	Kendall	RMSE	MSE	MAE
text−mining	0.456 (±0.03)	0.298 (±0.032)	0.205 (±0.021)	1.134 (±0.023)	1.305 (±0.054)	0.882 (±0.019)
GA	0.295 (±0.027)*	0.251 (±0.027)*	0.198 (±0.021)*	1.195 (±0.023)*	1.448 (±0.053)*	0.872 (±0.017)*
MRMR	0.394 (±0.026)	0.293 (±0.036)	0.23 (±0.029)*	1.141 (±0.027)*	1.33 (±0.066)*	0.846 (±0.026)*
RFE	0.367 (±0.03)	0.298 (±0.026)	0.237 (±0.02)*	1.145 (±0.021)*	1.326 (±0.047)*	0.827 (±0.019)*
cor−500	0.292 (±0.033)*	0.247 (±0.034)*	0.166 (±0.023)*	1.184 (±0.025)*	1.427 (±0.062)*	0.916 (±0.02)*
L1000−tm	0.247 (±0.036)*	0.191 (±0.031)*	0.13 (±0.021)*	1.242 (±0.025)*	1.564 (±0.061)*	0.954 (±0.019)*
L1000	0.282 (±0.026)*	0.22 (±0.027)*	0.148 (±0.019)*	1.207 (±0.018)*	1.47 (±0.043)*	0.933 (±0.016)*
var−500	0.278 (±0.034)*	0.241 (±0.024)*	0.162 (±0.016)*	1.207 (±0.021)*	1.474 (±0.052)*	0.935 (±0.018)*
var−100	0.226 (±0.027)*	0.187 (±0.021)*	0.124 (±0.014)*	1.248 (±0.017)*	1.569 (±0.043)*	0.955 (±0.016)*

Supplementary table S2. Mean and standard mean errors (SME) of the performance metrics for elastic net models based on various feature selection methods. Models with significantly different performance compared to the text-mining method are indicated with * (Student's T-test, $p < 0.05$)

Feature selection method	Pearson	Spearman	Kendall	RMSE	MSE	MAE
text−mining	0.465 (±0.024)	0.414 (±0.028)	0.29 (±0.021)	1.021 (±0.023)	1.062 (±0.048)	0.786 (±0.018)
GA	0.423 (±0.025)*	0.372 (±0.026)*	0.252 (±0.018)*	1.098 (±0.026)*	1.232 (±0.061)*	0.85 (±0.019)*
MRMR	0.234 (±0.028)*	0.327 (±0.024)*	0.223 (±0.016)*	1.055 (±0.026)*	1.139 (±0.057)*	0.818 (±0.022)*
RFE	0.208 (±0.023)*	0.31 (±0.028)*	0.213 (±0.02)*	1.095 (±0.025)*	1.221 (±0.055)*	0.855 (±0.021)*
cor−500	0.361 (±0.026)*	0.317 (±0.028)*	0.217 (±0.019)*	1.118 (±0.023)*	1.269 (±0.053)*	0.865 (±0.018)*
L1000−tm	0.366 (±0.024)*	0.304 (±0.031)*	0.209 (±0.022)*	1.114 (±0.021)*	1.258 (±0.048)*	0.866 (±0.017)*
L1000	0.383 (±0.026)*	0.33 (±0.026)*	0.226 (±0.018)*	1.097 (±0.023)*	1.223 (±0.051)*	0.848 (±0.018)*
var−500	0.343 (±0.027)*	0.31 (±0.025)*	0.212 (±0.018)*	1.132 (±0.025)*	1.305 (±0.053)*	0.874 (±0.019)*
var−100	0.301 (±0.027)*	0.259 (±0.027)*	0.173 (±0.018)*	1.169 (±0.023)*	1.387 (±0.053)*	0.918 (±0.02)*

Supplementary table S3. Mean and standard mean errors (SME) of the performance metrics for deep neural networks based on various feature selection methods. Models with significantly different performance compared to the text-mining method are indicated with * (Student's T-test, $p < 0.05$)

Feature selection method	Pearson	Spearman	Kendall	RMSE	MSE	MAE
text−mining	0.509 (±0.027)	0.441 (±0.029)	0.304 (±0.021)	0.159 (±0.013)	0.032 (±0.007)	0.133 (±0.013)
cor−500	0.477 (±0.025)*	0.407 (±0.028)*	0.281 (±0.02)*	0.164 (±0.014)*	0.035 (±0.008)*	0.139 (±0.014)*
L1000−tm	0.486 (±0.026)*	0.415 (±0.026)*	0.289 (±0.019)*	0.161 (±0.013)	0.033 (±0.007)	0.135 (±0.013)
L1000	0.475 (±0.026)*	0.405 (±0.027)*	0.282 (±0.02)*	0.163 (±0.014)*	0.034 (±0.008)*	0.138 (±0.014)*
var−500	0.282 (±0.027)*	0.225 (±0.024)*	0.153 (±0.017)*	0.153 (±0.012)*	0.029 (±0.006)*	0.129 (±0.012)*
var−100	0.17 (±0.024)*	0.158 (±0.025)*	0.11 (±0.019)*	0.16 (±0.013)	0.032 (±0.006)	0.136 (±0.013)*

Supplementary Table S4. Mean and standard mean errors (SME) of the performance metrics for random forest models based on various feature selection methods after removing common cell lines between CCLE and GDSE datasets. Models with significantly different performance compared to the text-mining method are indicated with * (Student's T-test, p < 0.05)

Feature selection method	Pearson	Spearman	Kendall	RMSE	MSE	MAE
text−mining	0.349 (±0.043)	0.313 (±0.048)	0.208 (±0.04)	1.105 (±0.03)	1.254 (±0.067)	0.864 (±0.025)
GA	0.311 (±0.029)*	0.292 (±0.03)	0.234 (±0.024)*	1.152 (±0.026)*	1.353 (±0.059)*	0.861 (±0.018)
MRMR	0.331 (±0.042)	0.307 (±0.044)	0.24 (±0.038)*	1.127 (±0.029)	1.299 (±0.07)	0.844 (±0.026)
RFE	0.361 (±0.028)	0.341 (±0.029)*	0.274 (±0.023)*	1.11 (±0.026)	1.256 (±0.056)	0.813 (±0.019)*
cor−500	0.278 (±0.048)*	0.239 (±0.049)*	0.154 (±0.039)*	1.163 (±0.034)*	1.395 (±0.079)*	0.914 (±0.029)*
L1000−tm	0.242 (±0.032)*	0.232 (±0.04)*	0.166 (±0.032)*	1.204 (±0.027)*	1.477 (±0.061)*	0.947 (±0.022)*
L1000	0.236 (±0.04)*	0.205 (±0.047)*	0.132 (±0.039)*	1.204 (±0.027)*	1.476 (±0.064)*	0.938 (±0.021)*
var−500	0.265 (±0.041)*	0.24 (±0.045)*	0.153 (±0.037)*	1.18 (±0.03)*	1.424 (±0.068)*	0.929 (±0.026)*
var−100	0.232 (±0.029)*	0.214 (±0.028)*	0.145 (±0.019)*	1.216 (±0.027)*	1.506 (±0.062)*	0.947 (±0.022)*

Supplementary table S5. Mean and standard mean errors (SME) of the performance metrics for elastic net models based on various feature selection methods after removing common cell lines between CCLE and GDSE datasets. Models with significantly different performance compared to the text-mining method are indicated with * (Student's T-test, p < 0.05)

Feature selection method	Pearson	Spearman	Kendall	RMSE	MSE	MAE
text−mining	0.438 (±0.044)	0.405 (±0.043)	0.291 (±0.034)	1.014 (±0.04)	1.087 (±0.071)	0.79 (±0.032)
GA	0.372 (±0.035)*	0.34 (±0.036)*	0.235 (±0.027)*	1.093 (±0.028)*	1.225 (±0.062)*	0.855 (±0.023)*
MRMR	0.426 (±0.032)	0.389 (±0.033)	0.273 (±0.023)	1.045 (±0.027)*	1.12 (±0.058)	0.813 (±0.022)*
RFE	0.403 (±0.029)*	0.378 (±0.029)*	0.259 (±0.022)*	1.069 (±0.025)*	1.165 (±0.053)*	0.838 (±0.021)*
cor−500	0.37 (±0.033)*	0.34 (±0.035)*	0.24 (±0.029)*	1.097 (±0.032)*	1.24 (±0.069)*	0.867 (±0.025)*
L1000−tm	0.372 (±0.033)*	0.327 (±0.041)*	0.234 (±0.033)*	1.092 (±0.031)*	1.228 (±0.064)*	0.859 (±0.024)*
L1000	0.379 (±0.034)*	0.328 (±0.042)*	0.236 (±0.033)*	1.082 (±0.036)*	1.217 (±0.067)*	0.844 (±0.027)*
var−500	0.312 (±0.044)*	0.289 (±0.032)*	0.196 (±0.022)*	1.139 (±0.029)*	1.328 (±0.065)*	0.899 (±0.027)*
var−100	0.315 (±0.031)*	0.281 (±0.028)*	0.189 (±0.019)*	1.146 (±0.03)*	1.347 (±0.066)*	0.908 (±0.023)*

References:

1. Fontaine, J.-F., Priller, F., Barbosa-Silva, A. & Andrade-Navarro, M. A. Génie: literature-based gene prioritization at multi genomic scale. *Nucleic Acids Res.* **39**, W455–W461 (2011).
2. Grünwald, V. & Hidalgo, M. Developing inhibitors of the epidermal growth factor receptor for cancer treatment. *J. Natl. Cancer Inst.* **95**, 851–867 (2003).
3. Carter, J. & Tadi, P. Erlotinib. in *StatPearls* (StatPearls Publishing, 2023).
4. Opdam, F. L., Guchelaar, H.-J., Beijnen, J. H. & Schellens, J. H. M. Lapatinib for Advanced or Metastatic Breast Cancer. *The Oncologist* **17**, 536–542 (2012).
5. Tsang, R. Y., Sadeghi, S. & Finn, R. S. Lapatinib, a Dual-Targeted Small Molecule Inhibitor of EGFR and HER2, in HER2-Amplified Breast Cancer: From Bench to Bedside. *Clin. Med. Insights Ther.* **3**, CMT.S3783 (2011).
6. Zhu, L. & Chen, L. Progress in research on paclitaxel and tumor immunotherapy. *Cell. Mol. Biol. Lett.* **24**, 40 (2019).
7. Kampan, N. C., Madondo, M. T., McNally, O. M., Quinn, M. & Plebanski, M. Paclitaxel and Its Evolving Role in the Management of Ovarian Cancer. *BioMed Res. Int.* **2015**, 413076 (2015).
8. Weaver, B. A. How Taxol/paclitaxel kills cancer cells. *Mol. Biol. Cell* **25**, 2677–2681 (2014).
9. Ferlini, C. *et al.* Paclitaxel directly binds to Bcl-2 and functionally mimics activity of Nur77. *Cancer Res.* **69**, 6906–6914 (2009).
10. Gavriilidis, P., Giakoustidis, A. & Giakoustidis, D. Aurora Kinases and Potential Medical Applications of Aurora Kinase Inhibitors: A Review. *J. Clin. Med. Res.* **7**, 742–751 (2015).
11. Martens, S. *et al.* RIPK1-dependent cell death: a novel target of the Aurora kinase inhibitor Tozasertib (VX-680). *Cell Death Dis.* **9**, 211 (2018).
